# Efficacy of magnetic resonance imaging in managing glycogen storage disease

**DOI:** 10.1186/s13023-025-03605-7

**Published:** 2025-03-27

**Authors:** Jhii-Hyun Ahn, Yong Whi Jeong, Yong Bok Choi, Yunkoo Kang

**Affiliations:** 1 Department of Radiology, Wonju Severance Christian Hospital, Yonsei University Wonju College of Medicine, Wonju, Korea; 2 Department of Medical Informatics and Biostatistics, Graduate School, Yonsei University, Wonju, Korea; 3 Department of Pediatrics, Wonju Severance Christian Hospital, Yonsei University Wonju College of Medicine, Wonju, Korea

**Keywords:** Magnetic resonance imaging, Glycogen storage disease, Hepatocellular adenoma, Liver, Risk prediction, Management strategies

## Abstract

**Background:**

Glycogen storage disease (GSD) is a rare genetic disorder requiring continuous management. It poses a risk of progression to hepatocellular adenoma (HCA) and hepatocellular carcinoma. While ultrasonography is the primary imaging modality to monitor liver health, it has limitations in assessing liver size and detecting HCAs, which can be addressed by magnetic resonance imaging (MRI). This study was conducted to evaluate the efficacy of MRI in the proactive management of GSD and its ability to predict HCA.

**Methods:**

This study included 32 patients with GSD from Wonju Severance Christian Hospital, of whom 29 underwent MRI examinations. Baseline characteristics, such as sex, height, weight, and body surface area (BSA), were recorded, along with laboratory markers. The MRI protocols included T2-weighted axial and coronal imaging, proton magnetic resonance spectroscopy, multi-echo Dixon imaging, magnetic resonance elastography, and T1 mapping. The correlation between liver volumes and laboratory results was analyzed, and logistic regression was used to analyze the association between the liver volume/BSA ratio and adenoma occurrence.

**Results:**

A significant correlation was observed between a high liver volume-to-BSA ratio and the likelihood of HCA development. Receiver operating characteristic curve analysis showed an area under the curve of 0.816 for predicting HCAs and a C-index of 0.847, indicating that MRI had high predictive accuracy. For each unit increase in the liver volume-to-BSA ratio, the probability of HCA increased by 1.005.

**Conclusion:**

MRI is valuable for assessing adenoma formation in patients with GSD. Although not intended for routine surveillance of all patients, MRI can be selectively used in high-risk cases to enable early detection and timely intervention, thereby reducing the risk of progression to malignant transformation.

## Background

Glycogen storage disease (GSD) is a rare genetic disorder characterized by severe hypoglycemia and various complications that require continuous management [1]. Effective monitoring of patients with GSD requires regular follow-up with laboratory tests and imaging studies. However, monitoring protocols for this disease lack established guidelines for identifying the most effective diagnostic tools [2, 3]. This lack of standardized protocols is particularly concerning because of the potential of GSD to progress to hepatocellular adenoma (HCA) and hepatocellular carcinoma (HCC), with some patients requiring liver transplantation [4–6]. Therefore, the accurate and continuous management of this condition is crucial.

Currently, ultrasonography is the primary imaging modality used to monitor liver health in patients with GSD. However, it has limitations in measuring liver size and assessing the overall condition, including the diagnosis of HCAs. Magnetic resonance imaging (MRI) is an objective tool for assessing liver health [7, 8]. MRI offers advantages such as the absence of radiation exposure and allows for advanced quantitative analyses, particularly for detecting focal liver lesions [9]. Despite these advantages, the use of MRI in managing patients with GSD is limited because of the lack of relevant research validating its efficacy.

The 2018 Practice Guidance by the American Association for the Study of Liver Diseases provides a comprehensive framework for diagnosing and managing HCC [10]. However, a major challenge in managing GSD lies in predicting the malignant transformation of hepatic adenomas to HCC in patients with GSD, owing to the lack of effective biomarkers. Existing biomarkers commonly used for liver cancer surveillance, such as α-fetoprotein (AFP) and carcinoembryonic antigen, often fail to indicate malignancy even in HCC cases, emphasizing the need for advanced imaging modalities [11, 12].

Given these challenges, evaluation of the efficacy of MRI as a proactive management tool in patients with GSD is essential. To address this, in the present study, we aimed to assess the efficacy of MRI in predicting the development of HCA in patients with GSD.

## Methods

This prospective study was approved by the Institutional Review Board of Wonju Severance Christian Hospital (WSCH), Yonsei University Wonju College of Medicine, Wonju, South Korea (approval number: CR321098).

### Subjects

A total of 32 patients with GSD undergoing regular follow-up at WSCH between 2021 and 2022 were included in this study. Routine follow-up included blood tests, ultrasonography, and FibroScan assessments. After obtaining informed consent, MRI scans were planned for 32 patients; however, three patients were excluded because of claustrophobia, leaving 29 patients for the final analysis.

### Clinical data

Baseline characteristics, including sex, height, weight, and body surface area (BSA), were recorded. Laboratory tests were performed to measure the levels of aspartate aminotransferase (AST), alanine aminotransferase (ALT), gamma-glutamyl transferase (GGT), alkaline phosphatase (ALP), lactate dehydrogenase (LDH), triglyceride (TG), cholesterol, and lactate.

### MRI

#### Protocol

Imaging was performed using a 3T MR system (MAGNETOM Skyra, Siemens Healthcare) equipped with a 30-channel body coil and 32-channel spine matrix coil. Patients were positioned supine, and all imaging was performed during expiratory breath-hold phases to minimize motion artifacts. The imaging protocol included:

i) Axial and coronal T2-weighted images were acquired using the following parameters: TR/TE, 1037 msec/80 msec; flip angle, 90°; matrix size, 324 × 235; and acquisition time, 3 min.
ii) Liver glucose content was measured using proton magnetic resonance spectroscopy (^1^H-MRS). A single voxel (20 × 20 × 20 mm³) was manually placed on the right liver lobe, avoiding the liver edges, visible blood vessels, and bile ducts. Raw magnetic spectral data were processed to calculate glucose content using a custom drawing tool (Singo.via, version VB30; Siemens).
iii) Multi-echo Dixon water-fat separation was performed to evaluate liver fat content. MRI-based proton density fat fraction (MRI-PDFF) maps, screening, and multi-echo Dixon reports were obtained using scanner reconstruction. Liver volumes were estimated.
iv) T1 mapping was performed using a prototypical Look-Locker sequence. For each acquired slice, the sequence involved continuous fast low-angle shot (FLASH) acquisition following an inversion pulse, which provided images at multiple inversion times. Three slices were obtained for each patient.
v) In magnetic resonance elastography (MRE), continuous longitudinal mechanical waves at 60 Hz were generated using an acoustic pressure driver device on the anterior chest wall. A prototype spin-echo echo-planar imaging sequence was acquired during breath-holding. In each patient, three sections were acquired in the axial plane (using the same slices as in T1 mapping).

#### Image analysis

Regions of interest (ROIs) were manually drawn on each section of the obtained T1 and MRE stiffness maps by a radiologist with nine years of experience. ROIs were carefully selected to include only the liver parenchyma, excluding areas immediately beneath the driver, bile ducts, blood vessels, and other regions with incoherent wave propagation. The corresponding magnitude, anatomical, and wave images were simultaneously evaluated. The measurements were restricted to areas where the confidence parameter of the stiffness reconstruction exceeded 95% [13, 14].

### Ultrasonography

To evaluate liver morphology, ultrasonography was performed using a commercial scanner (Aplio i800, Canon Medical Systems Corp.) equipped with a convex i8CX1 probe. The patients were scanned in the supine position, and echogenicity and the presence of focal lesions were evaluated using grayscale ultrasound.

### FibroScan

FibroScan was performed to measure liver stiffness and fat content in patients with GSD using transient elastography with a FibroScan device (Echosens, Paris, France). Liver stiffness measurement (LSM) values were expressed in kilopascals (kPa) and fat content was assessed using the controlled attenuation parameter (CAP). Multiple measurements were performed, and the median value of at least 10 valid LSM readings, with an interquartile range of less than 30% of the median, was used for subsequent analysis.

### Statistical analysis

Categorical variables are summarized as numbers and percentages, whereas continuous variables that follow a normal distribution are presented as means and standard deviations. Comparisons between MRI results were conducted using independent t-tests for parametric variables and the Wilcoxon rank-sum test for non-parametric data. Categorical variables were analyzed using chi-square or Fisher’s exact tests. Pearson’s correlation analysis was performed to determine the relationship between laboratory parameters and MRI results, as well as the relationship between liver MRI results and other diagnostic modalities. Univariate and multiple logistic regression analyses were performed to assess the association between the presence of HCA and the liver volume-to-BSA ratio and ^1^H-MRS glucose content. The goodness-of-fit of the model was confirmed using C-statistics. The optimal liver volume-to-BSA cutoff for predicting adenoma was determined using receiver operating characteristic (ROC) curve analysis. Statistical significance was set at *p* < 0.05. All analyses were performed using SAS 9.4 (SAS Institute, Cary, NC, USA) and R 4.0.3 (Institute for Statistics and Mathematics; http://cran.r-project.org).

## Results

### Basic characteristics

The basic characteristics of the 29 patients who underwent these examinations are summarized in Table 1.

**Table 1:** Summary of clinical and laboratory characteristics of the enrolled patients

Characteristics	Total (*N* = 29)	Type 1 (*N* = 27)	Type 3 (*N* = 1)	Unknown (*N* = 1)
Gender				
Male	18 (62.1)	17 (62.96)	0 (0.0)	1 (100.0)
Female	11 (37.9)	10 (37.04)	1 (100.0)	0 (0.0)
Age, years	15.14 (8.8)	15.00 (8.77)	26.0	8.0
Height, cm	144.92 (16.6)	145.31 (16.07)	161.0	118.4
Weight, kg	44.18 (13.7)	44.44 (13.97)	50.0	31.1
Protein	7.27 (0.6)	7.28 (0.65)	7.0	7.2
Albumin	4.81 (0.4)	4.83 (0.43)	4.2	4.8
AST	30.52 (14.8)	30.00 (14.86)	50.0	25.0
ALT	33.52 (28.1)	32.22 (26.94)	87.0	15.0
ALP	214.52 (109.7)	220.52 (109.23)	55.0	212.0
GGT	42.79 (29.6)	44.70 (29.84)	15.0	19.0
CK	108.90 (83.3)	96.67 (58.02)	429.0	119.0
LDH	185.55 (40.7)	182.15 (40.07)	229.0	234.0
Uric acid	7.45 (2.1)	7.63 (2.07)	5.5	4.4
Triglycerides	387.48 (378.8)	403.07 (07)	107.0	247.0
Total cholesterol	231.45 (51.5)	234.70 (51.46)	162.0	213.0
HDL cholesterol	46.97 (11.2)	46.78 (11.53)	45.0	54.0
LDL cholesterol	123.10 (42.7)	124.19 (44.04)	97.0	120.0
M2BPGi	0.62 (0.3)	0.62 (0.29)	0.5	0.8
Prealbumin	28.83 (5.8)	29.30 (5.70)	22.0	23.0
Liver volume	1.61 (0.6)	1.62 (0.56)	2.2	0.8
MR spectroscopy glucose	3772 (8,989)	4040 (9270)	302.0	2.3

Data are presented as n (%) or the mean (SD). AST, aspartate aminotransferase; ALT, alanine aminotransferase; ALP, alkaline phosphatase; GGT, gamma-glutamyl transferase; CK, creatine kinase; LDH, lactate dehydrogenase; M2BPGi, Mac-2 binding protein glycosylation isomer; MR: magnetic resonance

### Correlation coefficients for MRI and variables

**Fig. 1:**
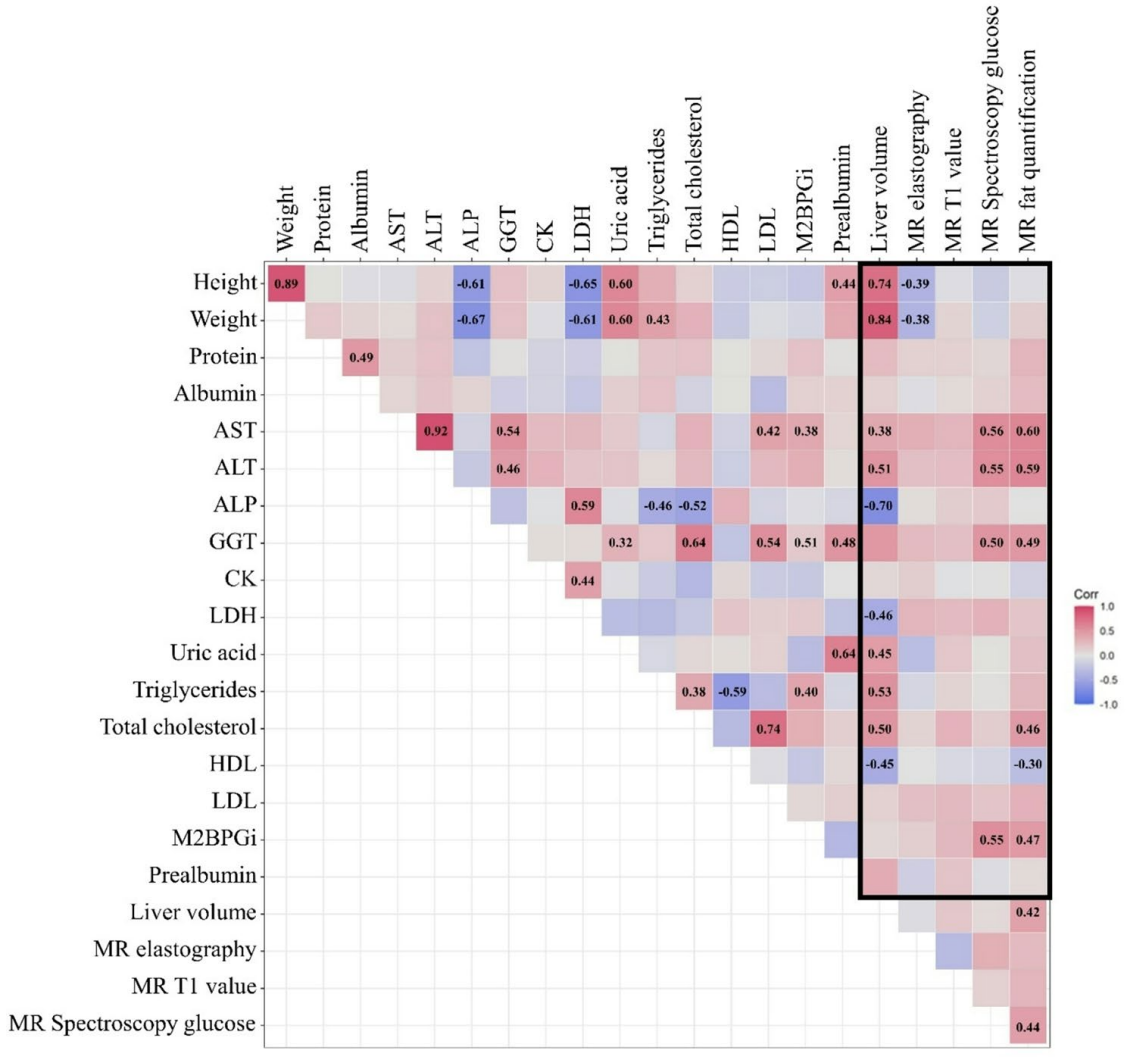
Correlation plot of the Pearson correlation coefficients of the MRI results

Estimating the correlations between the findings of laboratory tests and MRI scans revealed negative correlations between the liver volume and ALP levels. In addition, height, weight, and ALP levels were negatively correlated with LDH levels (Fig. 1). Conversely, liver volume was positively correlated with height, weight, uric acid, TG, and total cholesterol levels. Moreover, AST, ALT, GGT, and Mac-2 binding protein glycosylation isomer (M2BPGi) levels showed positive correlations with glucose levels from ^1^H-MRS and fat quantification measured using MRI-PDFF.

### Efficacy of MRI in predicting liver adenoma

**Fig. 2:**
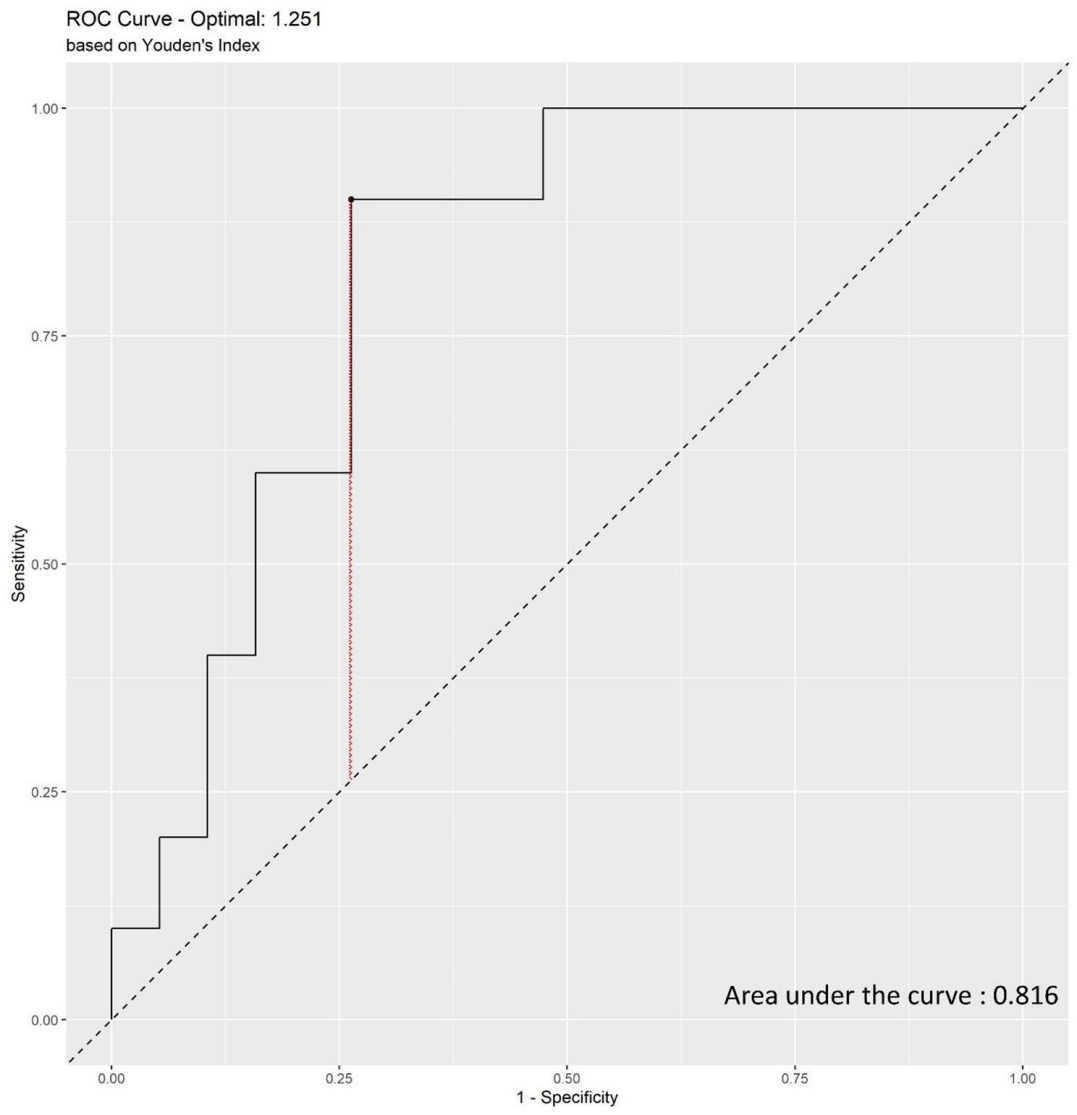
Receiver operating characteristic curve of liver volume to body surface area for predicting hepatocellular adenoma

The ROC curve used to determine the diagnostic performance of the liver volume-to-BSA ratio for detecting HCAs is shown in Fig. 2. The area under the curve (AUC) was 0.816, indicating good predictive accuracy. The optimal cutoff value of the liver volume-to-BSA ratio for the likelihood of HCA development was estimated to be 1.251.

Further analysis revealed that larger liver volumes relative to BSA were significantly associated with a higher likelihood of HCA development, with a C-index of 0.847, indicating high predictive accuracy (Table 2). Moreover, for each unit increase in the liver volume-to-BSA ratio, the odds of developing HCA increased by 1.005.

**Table 2:** Logistic regression for hepatocellular adenoma

	OR (95% CI)	
Liver volume-to-BSA ratio	1.005 (1.000–1.010)	
MR spectroscopy glucose	1.000 (1.000–1.000)	
C-index		

OR, odds ratio; CI, confidence interval; BSA,; MR,; C-index

### Comparative performance of liver MRI and other diagnostic modalities

Comparison of the liver MRI data with other diagnostic modalities revealed that the MRI-based liver volume was correlated with the FibroScan-derived CAP value (Table 3). Similarly, the MRI-based fat quantification value correlated with the ultrasound-based fat quantification and FibroScan-based CAP values.

**Table 3:** Correlation matrix of Pearson correlation coefficients

	MR fat quantification	MR spectroscopy glucose	Liver volume	US fat quantification	US shear-wave elastography	US shear-wave dispersion	Fibroscan (kPa)	Fibroscan (CAP)
MR fat quantification	1.000	0.438^*^	0.423^*^	0.653^**^	0.164	0.205	0.076	0.779^***^
MR spectroscopy glucose		1.000	0.064	0.162	0.058	0.090	0.119	0.251
Liver volume			1.000	0.102	0.120	0.016	0.289	0.630^**^
US fat quantification				1.000	0.188	-0.012	0.036	0.551^**^
US shear-wave elastography					1.000	0.744^***^	0.653^**^	0.083
US shear-wave dispersion						1.000	0.527^**^	0.148
Fibroscan (kPa)							1.000	0.153
Fibroscan (CAP)								1.000

*p < 0.05, **p < 0.01, ***p < 0.0001

## Discussion

In this study, we identified a significant positive correlation between liver volume relative to BSA and the likelihood of HCA development in patients with GSD. Specifically, a liver volume-to-BSA ratio of 1.251 or higher showed an association with an elevated risk of adenoma, suggesting the need for regular follow-up to prevent excessive liver enlargement. Proactive measures, such as dietary adjustments and controlled starch intake, should be considered to prevent liver enlargement.

Patients with GSD, particularly types I and III, face an increased risk of developing HCAs and HCC. In GSD type I (GSD I), the deficiency of glucose-6-phosphatase leads to the accumulation of glycogen and fat in the liver, resulting in hepatomegaly [3–5, 15]. Over time, chronic liver dysfunction and regenerative hyperplasia may lead to HCA formation, some of which progress to malignant transformation into HCC. The pathophysiology of these complications involves chronic metabolic imbalance caused by defective glycogen metabolism [16]. Specifically, GSD I leads to severe hypoglycemia, hyperlactic acidemia, hyperuricemia, and dyslipidemia, all of which contribute to hepatic and renal pathologies [4]. Effective management of GSD includes dietary management to maintain euglycemia, regular monitoring of liver and kidney function, and early intervention to address complications [17]. However, liver transplantation may be considered for patients with severe liver disease or a high risk of HCC [18].

A retrospective analysis by Kang et al. using the Korean National Health Insurance Service database revealed that 25 patients with GSD underwent liver transplantation, while 14 succumbed to complications [19]. These findings emphasize the severe complications associated with GSD, such as hepatic and renal dysfunction. Weinstein et al. have similarly highlighted the role of metabolic dysregulation in HCA formation in patients with GSD [20]. Despite these insights, predicting adenoma formation remains challenging, with current blood tests and imaging modalities being limited to detecting adenomas after their development. Recent advancements in biomarkers, as highlighted by Solhi et al. [21], offer promise for monitoring HCC but are inadequate for the early detection of HCAs in patients with GSD. Nevertheless, this study emphasizes the complementary role of MRI as a noninvasive and sensitive tool for early surveillance.

HCAs are inevitable in patients with GSD; however, recent studies have demonstrated that consistent management can decrease adenoma size. This study suggests that predicting adenomas before they appear allows for more proactive and effective management. Compared to ultrasonography, MRI offers superior sensitivity for detecting HCAs. Although FibroScan effectively measures liver stiffness, it lacks anatomical resolution, which is crucial for the comprehensive monitoring of patients with GSD. Moreover, while computed tomography (CT) can also measure liver volume, MRI is more beneficial for pediatric and long-term monitoring because it does not pose a radiation hazard [7]. MRI offers a significant advantage for patients requiring continuous monitoring by enabling the prediction and regular evaluation of potential HCA development. However, practical challenges, such as breath-holding and machine-related anxiety, may affect younger children. For instance, claustrophobia is reported in approximately 1% of the general population, and panic attacks occur in approximately 13% of patients undergoing MRI [22]. Moreover, MRI is generally feasible in patients aged 8 years and older without significant issues [], and none of the participants in this study experienced such challenges, further supporting the practical utility of MRI in this context.

Ultrasonography is widely used because of its accessibility and lack of radiation [23]. However, it is less effective in patients with hepatomegaly, which is a common feature of poorly managed GSD. Typically, if adenomas are detected by ultrasonography, only liver CT or MRI is used to assess their size and characteristics. MRI enables comprehensive assessment of liver size and structure, even in cases of significant enlargement. Furthermore, this study confirmed that the proactive use of MRI to monitor the BSA of patients and liver size to predict adenoma formation could be highly beneficial for managing patients with GSD. In situations where dietary control is inadequate and optimal metabolic control is poor, glycogen accumulates in the liver, causing liver damage that eventually leads to adenoma formation and, in the worst cases, HCC. Liver size has long been recognized as proportional to poor metabolic control in patients with GSD, a conclusion that does not inherently require advanced imaging modalities. Nevertheless, using MRI, we could assess the liver in its entirety and identify patients at risk of adenoma formation. This information is crucial for encouraging better metabolic management and preventing progression to more severe complications, such as HCC. Furthermore, it has been predicted that glycogen levels measured using MRS are related to HCA formation; however, in this study, we observed no such correlation, highlighting the need for further investigation.

The limitations of AFP and other biomarkers in detecting malignant changes in patients with GSD have been well documented. This study reaffirms the value of incorporating surveillance imaging, particularly MRI, to identify adenomas and their progression to malignancy. Early identification of HCAs through MRI can guide timely interventions and improve the outcomes of high-risk patients.

This study has several limitations. First, we did not observe the development of HCAs by prospectively performing MRIs. Because this was a cross-sectional study analyzing patients with and without liver adenomas, we could not determine the frequency of HCA development. Second, factors such as cornstarch intake, diet, and physical activity, which are known to influence glycogen accumulation and liver health, were not analyzed. Incorporating these variables into future studies could provide a more comprehensive understanding of their roles in adenoma formation. Third, the need for general anesthesia in younger patients due to motion artifacts and procedural anxiety remains a limitation of MRI. Although our findings suggest that MRI can be safely performed without sedation in many cases, its application should be carefully weighed against potential risks, and further studies should explore alternative noninvasive imaging modalities suitable for younger patients. Finally, we could not confirm whether optimal metabolic control reduced adenoma size in patients with GSD.

Despite these limitations, this study proposes a liver volume-to-BSA ratio cutoff value for predicting HCA formation in patients with GSD, offering an important tool for proactively managing these patients. These findings underscore the utility of MRI not only for comprehensive liver assessments but also for clinicians to identify at-risk patients and implement dietary and metabolic interventions earlier, potentially reducing the risk of progression to HCA and HCC.

## Conclusion

This study underscores the importance of advanced imaging techniques, such as MRI, in managing patients with GSD, especially for the early detection of adenomas and preventing malignant transformation. As biomarkers alone are often insufficient, MRI is a critical tool for comprehensive patient care. This study indicates that MRI can effectively and proactively manage patients with GSD. Further research is necessary to validate the cutoff values established in this study.

## Data Availability

The data supporting the findings of this study contain personal and confidential information. Due to privacy and ethical considerations, the data cannot be publicly shared. For further inquiries, please contact the corresponding author.

## Abbreviations

AFPα: Fetoprotein
ALP: Alkaline phosphatase
ALT: Alanine aminotransferase
AST: Aspartate aminotransferase
AUC: Area under the curve
BSA: Body surface area
CAP: Controlled attenuation parameter
GGT: Gamma-glutamyl transferase
GSD: Glycogen storage disease
GSD I: GSD type I
HCA: Hepatocellular adenoma
HCC: Hepatocellular carcinoma
LDH: Lactate dehydrogenase
LSM: Liver stiffness measurement
MRE: Magnetic resonance elastography
MRI: Magnetic resonance imaging
MRI-PDFF: MRI-based proton density fat fraction
M2BPGi: Mac-2 binding protein glycosylation isomer (M2BPGi)
ROC: Receiver operating characteristic
ROI: Regions of interest
TG: Triglyceride
WSCH: Wonju Severance Christian Hospital
